# CCL23 in Balancing the Act of Endoplasmic Reticulum Stress and Antitumor Immunity in Hepatocellular Carcinoma

**DOI:** 10.3389/fonc.2021.727583

**Published:** 2021-10-04

**Authors:** Dev Karan

**Affiliations:** Department of Pathology, Medical College of Wisconsin, Milwaukee, WI, United States

**Keywords:** tumor microenvironment, endoplasmic reticulum, hepatocellular carcinoma, antitumor immunity, chemokine ligand CCL23

## Abstract

Endoplasmic reticulum (ER) stress is a cellular process in response to stress stimuli in protecting functional activities. However, sustained hyperactive ER stress influences tumor growth and development. Hepatocytes are enriched with ER and highly susceptible to ER perturbations and stress, which contribute to immunosuppression and the development of aggressive and drug-resistant hepatocellular carcinoma (HCC). ER stress-induced inflammation and tumor-derived chemokines influence the immune cell composition at the tumor site. Consequently, a decrease in the CCL23 chemokine in hepatic tumors is associated with poor survival of HCC patients and could be a mechanism hepatic tumor cells use to evade the immune system. This article describes the prospective role of CCL23 in alleviating ER stress and its impact on the HCC tumor microenvironment in promoting antitumor immunity. Moreover, approaches to reactivate CCL23 combined with immune checkpoint blockade or chemotherapy drugs may provide novel opportunities to target hepatocellular carcinoma.

## Introduction

Hepatocellular carcinoma (HCC) is a basic form of liver malignancy and is the leading cause of liver cancer deaths worldwide. In 2021, approximately 43 000 new cases will be diagnosed, with an estimated 30 000 deaths in the United States (1). Like with any other cancer type, HCC development is a multistep process involving hepatic cell injury, chronic infection, fibrosis, cirrhosis, and finally, liver cancer. Understanding this process has helped identify new genetic/molecular drivers and develop treatment approaches. Thus far, the five-year survival expectancy rate for liver cancer is only ~18% and is further reduced to ~15% in black patients (1). Treatment with chemotherapy drugs has low efficacy due to the emergence of drug-resistance disease. For advanced HCC, systemic therapy with sorafenib, lenvatinib, or regorafenib delivers limited survival benefits (2, 3). Unfortunately, liver cancer incidence has increased rapidly (2 to 3% annually) in the United States, with a ~43% increase in HCC-related mortality over the past two decades. Besides, a projected surge in death rates of ~57.6% (6.6 to 10.4) from 2015 to 2035 is very alarming (2). Thus, understanding the biological process of HCC will help to create new and effective treatment opportunities.

As a vital organ, the liver tissue engages in essential physiological activities, adding new challenges to understanding liver oncogenesis. However, based on evolutionary history and the process of HCC development, the most common genetic and epigenetic alterations include mutations in the TERT promoter, TP53, β-catenin, AXIN1, ARID1A, ARID2, CDKN2A, and CCND1 genes (4–6). Here, we describe a novel perspective of a CC-chemokine ligand (CCL23) in context with HCC tumor development and its potential in mitigating the ER stress leading to remodeling the HCC tumor microenvironment in favor of enhanced antitumor immunity.

## Functional Analysis of CCL23 in Cancer and Non-Cancerous Diseases

In general, chemokines contribute to a miscellany of biological activities during the process of HCC development, helping tumor cells evade the immune system and tumor angiogenesis, invasion, and metastasis (7). CCL23, also known as myeloid progenitor inhibitory factor-1, suppresses the production and release of polymorphonuclear leukocytes and hematopoietic progenitor cells from the bone marrow (8, 9). CCL23 is considered a relatively new chemokine, and its role in tumor cell progression, metastasis, and other disease conditions is not well characterized, instead primarily limited to correlated expression analysis. Several disease types – including acute myeloid leukemia, ischemic strokes, coronary atherosclerosis, chronic kidney disease, and systemic mastocytosis – have indicated expression of CCL23 correlated with disease progression (10–14). A few studies on cancer types have primarily demonstrated CCL23 gene expression in tissues without much attention to biological or molecular functions. For example, a high throughput genomic data analysis in ovarian cancer revealed CCL23 as one of the candidate genes associated with ovarian cancer (15). Colorectal studies displayed somewhat ambiguous observations. A protein-based array of 507 targets in six samples of colorectal patients revealed an upregulation of CCL23 in rectal cancer compared to non-rectal cancer (16). Conversely, inflammatory gene expression analysis in colorectal patients showed a reduced level of CCL23 in adenoma and adenocarcinoma than in normal mucosa (17). Furthermore, a meta-analysis of 1577 breast cancer patients from the Oncomine datasets revealed a negative correlation of CCL23 and its receptor CCR1 with metastasis-free survival. However, this association was limited to HER2^+^ breast cancer patients and did not persist in HER2^-^ patients or other subtypes (18).

The mechanistic and functional studies on CCL23 are hampered because, as such, there is no defined murine CCL23. However, genomic analysis of CC chemokines suggested that the chemokine ligand CCL6 is the mouse homolog of the human CCL23 (19). Studies in a BCR-ABL-induced leukemia murine model showed that CCL6 was required for an interferon consensus sequence-binding protein (ICSBP)-induced, vaccine-like immunoprotective effect. Normal cells expressed high levels of CCL6 compared to leukemic cells, but IFN-α treatment could reactivate the expression of CCL6 in leukemic cells. In this mouse model, shRNA-directed CCL6 knockdown in mouse tumor cells abrogated the anti-leukemic survival response generated by ICSBP, suggesting the role of CCL6 as a potential immunomodulatory chemokine (20). Clinical extension of these studies revealed that chronic myeloid leukemia (CML) patients who responded to IFN-α treatment showed higher levels of CCL23 compared to non-responders. Similarly, increased CCL23 was observed in patients with multiple myeloma, melanoma, and renal cell carcinoma after exposure to IFN-α (20).

Mechanistic studies in mouse models further demonstrated that CCL6 promotes innate immunity *via* immune cell activation and serves as a chemoattractant for CD11b^+^, IFN-producing dendritic cells, NK cells, and CD4^+^ T cells (20, 21). Therefore, decreasing CCL23 expression in leukemic cells could be a mechanism for CML cells to escape from the immune system. In addition, an *in vitro* study on freshly prepared eosinophils from human PBMCs showed a slight increase in CCL23 mRNA following 6 hours of IFN-α treatment (22).

In a breast cancer mouse model, a protein array analysis in the lungs of 4T1 tumor-bearing mice showed a higher level of CCL6 along with IL-33, CCL12, CCL17, and MMP-9 than in normal mice. Treatment with *Cordyceps sinensis* (a Chinese herb) reduced 4T1 tumor cell metastasis to the lungs associated with a decrease in IL-33, CCL17, and MMP-9 (23). However, the level of CCL6 chemokine remains unaltered and warrant further investigation for its role in the anti-metastatic activity of cancer cells.

## CCL23 in Hepatocellular Carcinoma

**Figure 1:**
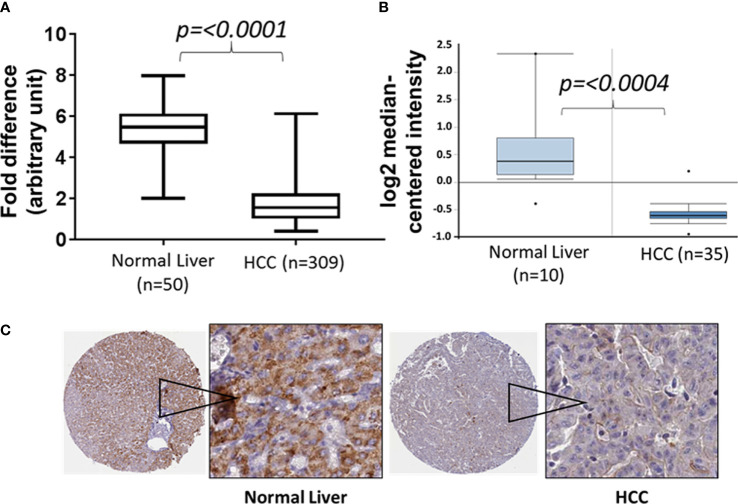
Box and Whisker plots of CCL23 transcript expression levels from two different data sets (**A**: TCGA liver cancer and **B**: Oncomine) and **(C)** the expression of CCL23 protein by immunohistochemistry data sets from the Human Protein Atlas (proteinatlas.org) in the normal liver and hepatocellular carcinoma (HCC). The expression level of CCL23 is significantly lower in HCC as compared to the normal liver.

The Cancer Genome Atlas (TCGA) data sets have significantly helped in mining the genomic landscape of human diseases and in elucidating the role of the tumor microenvironment (TME) both from the perception of tumor cells and molecular subtyping of tumor-infiltrating lymphocytes (24). Nonetheless, TCGA data sets continue to offer many opportunities in identifying new molecular drivers, mutational analyses, gene signaling pathways, and diagnostic/prognostic biomarkers for various cancer types. We extracted the mRNA expression profile from a TCGA data set (TCGA Liver Cancer) for normal tissues and primary tumors to elucidate the potential role of CCL23 in association with the immunobiology of hepatocellular carcinoma (25). Analysis of the data set from the cohort TCGA Liver Cancer showed a significantly lower (*p = 0.0001*) expression of the CCL23 transcript in HCC compared to normal liver tissue (**Figure 1A**). Extension of this analysis to the Oncomine data set also revealed significant downregulation (-2.15 fold; *p = 0.0004*) of CCL23 in HCC compared to the normal liver (**Figure 1B**) (26). In support of these observations, we examined the Human Protein Atlas resources for CCL23 protein expression (v20.proteinatlas.org/ENSG00000274736-CCL23/pathology/liver+cancer). The immunohistochemistry (IHC) data revealed a low CCL23 protein level in HCC compared to the normal liver (**Figure 1C**). This data set comprises 12 HCC samples; CCL23 protein was not detectable in eight (8/12), showed low expression levels in three (3/12), and displayed a moderate level of CCL23 protein expression in one (1/12). In these small numbers of samples from the protein atlas data set, we also noticed that CCL23 protein expression in the normal liver of females was comparatively higher than males. These observations suggest a lower level of CCL23 in HCC at both the mRNA and protein levels than normal liver tissue.

**Figure 2:**
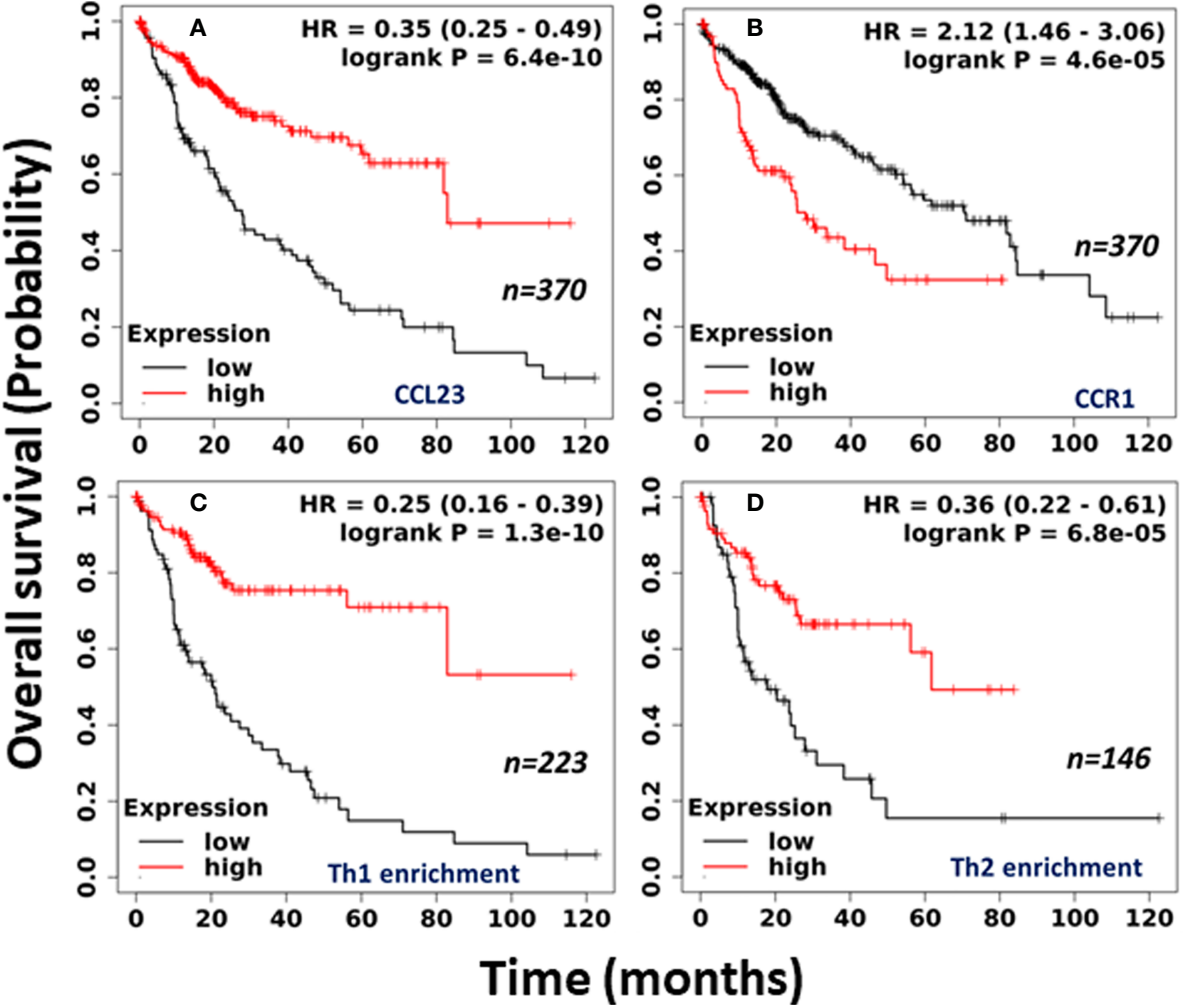
Clinical association of **(A)** CCL23; **(B)** CCR1; **(C)** Th1 cell enrichment; and **(D)** Th2 cell enrichment in human HCC tissues with overall survival. (Data source: Kaplan-Meier Plotter, Pan-cancer RNA-seq).

We further selected mRNA (RNA-seq) liver cancer data sets from Kaplan Meier (KM) Plotter for the pan-cancer and examined the correlation between CCL23 mRNA and the survival probability (27). The analysis includes all stages, grades, both sex and race, and risk factors. The cutoff values used in the analysis are based on auto best cutoff values. KM Plotter analysis revealed a significantly poor (*p = 6.4x10^-10^*) prognosis in HCC patients having low levels of CCL23, with a median survival of 27.57 months vs. 82.87 months (**Figure 2A**). However, the expression of CCL23 receptor CCR1 revealed an opposite trend – high CCR1 expression was associated with shorter survival in HCC patients (**Figure 2B**). This seemingly conflicting observation for CCR1 is not surprising in light of the ambiguity regarding the functional activities of chemokine receptors. CCR1 serves as a receptor for multiple chemokines and may not necessarily follow a similar functional correlation as CCL23.

To understand the clinical significance between the low level of CCL23 and the infiltrated immune cells, we further examined the immune cell composition within the HCC tissues and its impact on overall survival. Surprisingly, in association with low levels of CCL23, enrichment of the human HCC tissues with type 1 T-helper (Th1) cells revealed significantly shorter (*p =* 1.3x10^-10^) median survival of 9.3 months vs. 56.17 months in HCC patients (**Figure 2C**). Enrichment with type 2 T-helper (Th2) cells also showed significantly reduced (*p =* 6.8x10^-5^) overall survival with low levels of CCL23, but the median survival was better than the Th1 cell enrichment (17.83 months vs. 61.73 months; **Figure 2D**). Furthermore, mechanistic studies on the role of T-helper cells showed that liver Th1 cells might drive the infiltration of myeloid-derived suppressor cells (MDSCs) in the inflamed TGF-β1 knockout mouse liver, leading to diminished survival (28).

On the other hand, Th2 cytokines IL-4 and IL-13 regulate CCL23 expression in CD14^+^ monocytes (29). Human neutrophils, which do not produce CCL23 in response to Th2 cytokines, express and release CCL23 upon stimulation with toll-like receptor agonists (Resiquimod and LPS) or TNF-α, suggesting the role of CCL23 in driving the recruitment of immune cells at the inflamed site in favor of a controlled immune response (30). Although the infiltration of Th1 immune cells in the HCC TME is linked with a favorable clinical response (31), in the absence of CCL23, Th1 cells may not exert cytolytic function. Also, the operational support of Th1 cells may be outweighed due to an anergic or exhausted cytotoxic immune cells ecosystem within the HCC tumor microenvironment due to a low level of CCL23. However, additional studies are essential to validate the infiltration of Th1 and Th2 immune cells in HCC tissue, predicting the survival benefit associated with the loss of CCL23.

Lu et al. performed bioinformatics analysis of CCL23 in human HCC tissues from multiple online databases (GEPIA, HCCDB, MetaScape, TIMER, TISIBD, and KM Plotter) and revealed low expression of CCL23 in all stages of HCC in association with poor prognosis (32). The gene ontology function and Kyoto gene and genome encyclopedia pathway of CCL23 co-expressed gene in HCC were enriched in immune cells and mainly associated with CD8^+^ T cells and macrophage activation (32). Jia-Jie et al. analyzed CCL23 expression in liver cancer and adjacent normal liver tissues from 196 cases of radical hepatectomy by real-time fluorescence quantitative-PCR and 82 HCC tissues with matched cancer and adjacent normal tissues by IHC. The multivariate Cox regression analysis revealed a significantly lower expression of CCL23 in liver cancer tissues compared to adjacent normal. The liver cancer patients with higher CCL23 expression showed better survival than those with low CCL23 (33).

Collectively, these observations suggest a critical role of CCL23 in HCC. The presence of CCL23 may help create a tumor-suppressive environment by recruiting leukocytes to the tumor site and postulate that the loss of CCL23 serves as a driver in the oncogenesis of hepatic tumor cells. Therefore, determining the onco-immunologic function of CCL23 may help to understand the process of HCC development better.

## ER Stress and Immune Suppression in HCC

The endoplasmic reticulum is a vital cell organelle engaging in multiple physiological functions, including protein folding and transport of the synthesized proteins. During the process of tumor development, ER stress leads to the activation of the unfolded protein response (UPR), distressing cellular metabolic activities regulating various intracellular functions, including protein folding, calcium homeostasis, lipid metabolism, cell differentiation, and protein translocation (34, 35). UPR is an adaptive cellular mechanism to counteract the accumulated protein misfolding stress, ultimately evolving anti-apoptotic and drug-resistance machinery of tumor cells. In the absence of stress, ER chaperone glucose‐regulated protein 78 (GRP78) binds to UPR and sequesters the UPR sensors protein kinase R-like ER kinase (PERK), inositol‐requiring enzyme 1α (IRE1α), and transcription factor 6 (ATF6).

Various studies document that ER stress and UPR signaling pathways contribute to nearly all forms of acute and chronic liver diseases and promote HCC cell survival, proliferation, and angiogenesis (36–38). The expression levels of UPR signaling proteins IRE1α, XBP1s, PERK/ATF4, CHOP, and ATF6 are increased in HCC model systems and are associated with HCC growth and development (39–41). Interestingly, UPR kinetic studies in HCC revealed that IRE1α is activated during tumor initiation and the PERK pathway during tumor progression, while ATF6 is only moderately activated in developed tumors (39). HCC biopsies from human patients showed elevated XBP1 expression levels, whereas cancer cells deficient in XBP1 are less prone to developing solid tumors in nude mice (42). In an orthotopic mouse model of HCC, the PERK inhibitor significantly reduces the tumor burden by killing ER-stressed HCC cells (39). Similarly, liver-specific knockout IRE1α mice fed with a regular diet showed low diethylnitrosamine-induced HCC and suppressed HCC progression in mice fed with a high-fat diet. The tumor growth inhibition was associated with decreased hepatocyte proliferation, STAT3 activation, and reduced tumor-promoting inflammatory cytokines TNF-α and IL-6 (40).

An increased in GRP78 expression level is reported as a pro-survival factor for cells undergoing ER stress. In HCC tumors from patients treated with sorafenib, 73% showed high GRP78 expression, which was associated with the shortest progression-free survival (41). Elevated GRP78 is also proposed as a predictive biomarker in HCC patients treated with sorafenib (43). Moreover, GRP78 is linked with activation of the Wnt/catenin pathway in HCC (44). Higher GRP78 is positively correlated with Golgi protein 73 (GP73) and a high density of tumor-associated macrophages displaying CD206 expression leading to poor prognosis in HCC patients (45). The increased expression level of CD147 in HCC also serves as a UPR inducer, promoting ER stress and HCC cell survival, decreasing the efficacy of the chemotherapy drug adriamycin in animal studies (37, 46). Indeed, chemotherapy drug-induced cellular stress in cancer cells leading to adaptation, and drug-resistant cancer cells occur frequently in HCC.

Hepatocellular carcinoma is inflammation-associated cancer and usually progresses on the pretext of inflammation in the liver (47). The chronically inflamed tumor microenvironment is characterized by a high degree of ER stress, activating UPR signaling, and immune suppression within tumors (48, 49). Any of the UPR sensors can trigger NF-κB-inducing, tumor-promoting, pro-inflammatory cascade implicated in macrophage activation and TNF-α, IL-6, IL-1β, and IL-8 cytokines production (50–52). Similarly, vitamin D receptor deficiency leads to persistent UPR activation, promotes hepatic macrophage infiltration, and produces pro-inflammatory cytokines (53). In addition, inflammatory mediators can maintain and augment ER stress in the inflamed tissues, providing a feedback mechanism, attributing to the plasticity of macrophage polarization at the tumor site. Altogether, UPR is known to associate with cancer initiation, tumor cell quiescence and aggressiveness, EMT, angiogenesis, autophagy, and a metabolic switch in cancer cells to adapt to the challenging, stressful TME and supporting immunosuppression (37, 49, 54).

The degree of tumor-infiltrating lymphocytes in HCC is closely associated with tumor recurrence. High levels of MDSCs and T-regulatory cells in patients are reportedly related to aggressive HCC and low survival rates (55–57). Patients infected with the hepatitis-C or hepatitis-B virus accumulate T-regulatory cells in HCC tumor tissues involving TGF-β signaling (58). There are multiple mechanisms linked to the increased association of immune suppressor cells in HCC. However, emerging evidence suggests that ER stress promotes the escape of tumor cells from immune surveillance and hampers antitumor immunity through the regulation of immunosuppression (54, 59–61). UPR and the increased level of inflammatory cytokines by tumor cells could be transmitted into bone marrow-derived dendritic cells – such distressed DCs exhibit impaired antigen presentation and cross-priming CD8^+^ T cell, supporting enhanced tumor growth in mice (62). Increased ER stress-related proteins were also correlated with M2 macrophage recruitment and PD‐L1 expression in HCC tissues (63). Targeting MDSCs has been shown to enhance the therapeutic efficacy of sorafenib and immune checkpoint inhibitors in HCC murine models. Reversing the pro-tumor effects of MDSCs could be achieved by depleting MDSCs, blocking MDSC trafficking into TME (64, 65). CCL23 functionally contributes to the modulation of the immune response by promoting leucocyte trafficking as well as directing the migration of monocytes, macrophages, dendritic cells, and T lymphocytes to the sites of injury or infection (66, 67). Therefore, manipulation with CCL23 may help reprogram the HCC TME by targeting myeloid checkpoints to harness the power of antitumor immunity with immune checkpoint inhibitors or other forms of immunotherapy approaches.

## CCL23-Directed ER Stress Mitigation

A high level of ER stress is closely linked to immune responses and activates pro-inflammatory cytokines/chemokines. For example, increased CXCL10 (IP10) following liver graft injury leads to ER stress-associated cisplatin-resistant HCC, and the neutralization of CXCL10 sensitizes cisplatin treatment, which suppresses tumor growth (68). A study in type 2 diabetic mouse model showed that inhibiting chemokine receptor 2 (receptor for CCL2) improves hepatic steatosis by reducing ER stress and inflammation (69). Additionally, visfatin-induced hepatic inflammation in a mouse model of methionine-choline-deficient diet showed increased CXCL2, CXCL8, and MCP-1 associated with ER stress upregulation (70). On the other hand, in a breast cancer study, tunicamycin-activated ER stress increases the endogenous level of CCL5 mRNA and protein and prevents MCF-7 cells migration (71). This study supports our observations that approaches to reactivate endogenous CCL23 may help repress hepatic tumor cell growth associated with reduced ER stress.

A bovine endometrial cell model showed that tunicamycin (an antibiotic)-induced ER stress inhibits epithelial cell proliferation (72). Addition of CCL23 rescued cell proliferation by activating PI3K/AKT and MAPK signaling while simultaneously reducing the expression levels of UPR-signaling proteins, including PERK, IRE1α, and ATF6. Moreover, CCL23 inhibited the expression of LPS-induced, pro-inflammatory cytokines TNF-α, IL-6, and IL-8 and restored intracellular Ca2^+^ levels required for optimal ER functioning. This observation corroborates with the HCC growth inhibition in liver-specific knockout IRE1α mice. In addition, CCL23 treatment restored the basal level of tunicamycin-induced downstream molecules (eIF2α and CHOP) and the ER chaperone protein (GRP78). A similar phenomenon of CCL23-induced ER stress alleviation was observed in porcine endometrial luminal epithelial cells (73). Thus, the CCL23 chemokine likely has a significant potential to alleviate ER stress by rescuing the UPR-sensors and reducing the pro-inflammatory cytokine profile, diminishing the immunosuppressive HCC tumor microenvironment and enhancing antitumor immunity.

## Conclusions and Future Perspectives

ER stress is known to impair the immune cell function, promote MDSCs, T-regulatory cells, and M2 macrophages with an overall impact on immune evasion. Even though HCC is a deeply enriched site of immune cell engagement, the commonly used immune checkpoint inhibitor-based therapies showed limited success. ER stress or viral infection is associated with increased PD-L1 expression in HCC tissues, yet the PD-1 inhibitor nivolumab showed a ~15% response rate in HCC while the CTLA4 blockade showed a relatively lesser objective response (74–76).

**Figure 3:**
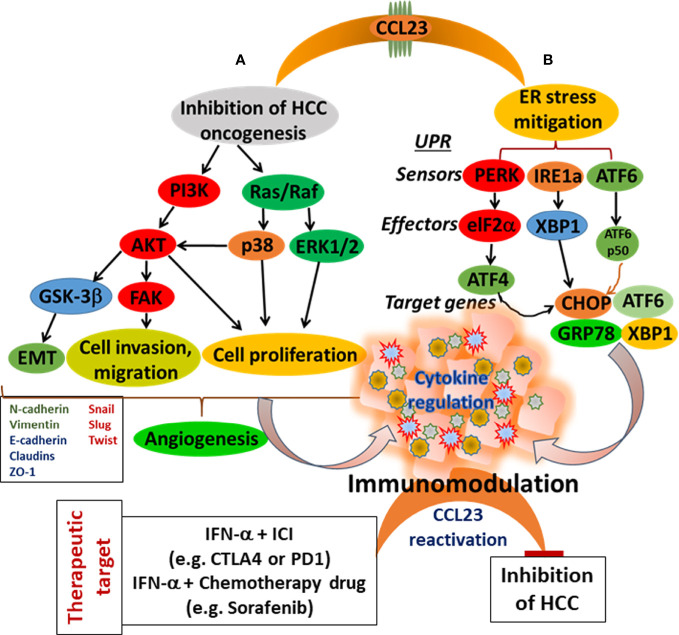
Schematic presentation of CCL23 functions for the modulation of the HCC tumor microenvironment. **(A)** Targeting cellular signaling cascade inhibiting HCC cell progression, and **(B)** Mitigating of ER stress. Both events lead to immunomodulation with a potential to enhance antitumor immunity.

This review accentuates the observations that low expression of CCL23 in HCC tissues is associated with the poor prognosis of HCC patients. Therefore, to understand the significance of CCL23 in HCC oncogenesis, we hypothesize two potential scenarios: 1) CCL23 might play a vital role in reducing ER stress and help recruit macrophages and dendritic cells, augmenting antitumor immunity; and 2) expression of CCL23 may serve as a tumor suppressor by inhibiting the invasive features (such as hepatic tumor cell invasion and migration). These molecular events will ultimately influence the TME and may rejuvenate immune cell functions by reducing the function and frequency of immune suppressor cells (**Figure 3**). Following CCL23-directed reprogramming of the TME, HCC may be amenable to immune-based therapies, including checkpoint blockade inhibitors to enhance the therapeutic efficacy. For example, since IFN-α induces antitumor immunity *via* CCL23 reactivation in humans, a combinatorial approach of IFN-α and checkpoint blockade inhibitors may provide a novel opportunity targeting hepatocellular carcinoma (**Figure 3**). These observations may inspire a new direction of research investigating the cross-talk between ER stress and immunomodulation in the HCC tumor microenvironment using CCL23 as a molecular target. Preclinical validations of this potential concept may provide much-needed information to improve clinical management of hepatocellular carcinoma.

## Author Contributions

The author confirms being the sole contributor of this work and has approved it for publication.

## Conflict of Interest

The author declares that the research was conducted in the absence of any commercial or financial relationships that could be construed as a potential conflict of interest.

## Publisher’s Note

All claims expressed in this article are solely those of the authors and do not necessarily represent those of their affiliated organizations, or those of the publisher, the editors and the reviewers. Any product that may be evaluated in this article, or claim that may be made by its manufacturer, is not guaranteed or endorsed by the publisher.
